# 高危冒烟型多发性骨髓瘤的风险分层及治疗进展

**DOI:** 10.3760/cma.j.cn121090-20240416-00144

**Published:** 2024-12

**Authors:** 洁琼 周, 牧 郝, 刚 安

**Affiliations:** 1 中国医学科学院血液病医院（中国医学科学院血液学研究所），血液与健康全国重点实验室，国家血液系统疾病临床医学研究中心，细胞生态海河实验室，天津 300020 State Key Laboratory of Experimental Hematology, National Clinical Research Center for Blood Diseases, Haihe Laboratory of Cell Ecosystem, Institute of Hematology & Blood Diseases Hospital, Chinese Academy of Medical Sciences & Peking Union Medical College, Tianjin 300020, China; 2 天津医学健康研究院，天津 301600 Tianjin Institutes of Health Science, Tianjin 301600, China

## Abstract

冒烟型多发性骨髓瘤（SMM）患者是一个异质性群体，不同患者预后不一，部分高危患者2年内有较高风险向多发性骨髓瘤（MM）转化。高危患者的定义在不同风险模型间一致性不高，各种生物标志物与新技术的结合改善了风险模型的预测效能，临床研究中往往组合应用不同的风险模型。治疗MM的新药逐渐被应用于高危SMM的治疗中，单药或双药治疗延长了患者的无进展生存期，但多数研究尚未显示患者的总生存获益。多药联合、强化治疗能够使高危SMM实现深度缓解，但是多为单臂研究，尚不足以证明多药治疗疗效优于较低强度治疗，仍需进一步探索高危SMM患者的治疗方案。本文对高危SMM的风险分层及治疗进展进行综述。

## Abstract (en)

Smoldering multiple myeloma（SMM）patients are a heterogeneous group with variable prognosis. A subset of SMM patients have a higher risk of progressing to multiple myeloma（MM）within 2 years. The definition of high-risk patients is not consistent among different risk models, and the combination of various biomarkers and new technologies improves the predictive performance of risk models. In clinical research, different risk models are often combined and applied. New drugs for treating MM are gradually being applied in the treatment of high-risk SMM. Single or dual drug therapy prolongs the progression free survival of patients, but most studies have not yet shown an overall survival benefit for patients. Multi drug combination and intensive treatment can achieve deep relief in high-risk SMM, but most of the studies are single arm studies, which are not yet sufficient to prove that multi drug treatment is more effective than lower intensity treatment. Further exploration of treatment plans for high-risk SMM patients is still needed. This article reviews the progress in risk stratification and treatment of high-risk SMM.

冒烟型多发性骨髓瘤（smoldering multiple myeloma，SMM）是一种介于意义未明单克隆丙种球蛋白血症（monoclonal gammopathy of undetermined significance, MGUS）和活动性多发性骨髓瘤（MM）之间的无症状恶性克隆性浆细胞疾病。2014年，国际骨髓瘤工作组（IMWG）将一部分根据生物标志物定义的超高危SMM归入MM中，SMM现在仅包括血清M蛋白≥3 g/dl或24 h尿M蛋白≥500 mg和（或）骨髓中异常浆细胞（aBMPC）比例≥10％（但不超过60％）且没有骨髓瘤定义事件（MDE）的患者[1]。SMM在40岁及以上人群中的患病率为0.53％，在诊断后的前5年内以每年约10％的速度发展为MM，随后5年以每年3％的速度发展，之后以每年1.5％的速度发展[2]–[3]。既往基于对药物不良反应和疗效的考虑，对SMM患者采取“观察等待”策略[4]–[5]。然而SMM患者预后不一，大部分患者疾病进展缓慢，甚至终身没有进展，有小部分患者疾病进展较快，在2年内高风险向MM转化[6]。对于这部分高危SMM患者的管理存在较多争议，国际上尚未就这部分患者的最佳治疗方案达成共识。本文就高危SMM患者危险度分层及治疗相关进展进行综述。

一、SMM风险分层模型的演变

SMM患者预后不一，治疗的前提是识别SMM患者中的高危群体，尽可能避免过度治疗或治疗不足。目前SMM风险分层的研究主要有两个方向：研发准确度高且结果稳健的模型、将更多预后标志物添加到现有预后模型中以改善其效能。

1. 经典SMM风险分层模型：2007年，西班牙骨髓瘤工作小组推出国际上首个SMM风险分层模型——PETHEMA模型，该模型识别了2个高危因素，1个是通过流式细胞术检测到aBMPC在总浆细胞中所占比例不低于95％，另1个是免疫不全或免疫麻痹，同时存在这2个高危因素为高危SMM，仅1个高危因素为中危SMM，无高危因素为低危SMM[7]。作为首个SMM预后模型，PETHEMA模型在研究中得到了广泛应用，然而，该模型依赖流式细胞术检测异常浆细胞，限制了其在临床实践中的应用。

2008年，梅奥诊所发现SMM患者血清游离轻链（serum free light chain, sFLC）比例与患者预后相关，在此基础上推出Mayo 2008模型，血清中M蛋白峰值≥3 g/dl、aBMPC比例≥10％、sFLC比例≥8或≤0.125为高危因素，同时存在3个高危因素者为高危SMM，存在2个高危因素者为中危SMM，≤1个高危因素者为低危SMM。Mayo 2008模型采用临床指标对患者进行预后分层，较PETHEMA模型更具有临床实践意义，得到了广泛应用。2010年IMWG更新了对SMM患者的管理策略，首次建议对所有SMM患者进行风险分层[8]。PETHEMA模型和Mayo 2008模型极大促进了SMM相关临床研究的开展。在上述2个模型发表之前开展的临床研究并未对SMM患者进行风险分层，造成了潜在的中低危患者治疗过度和高危患者治疗不足，可能是沙利度胺等药物治疗组和对照组患者生存无差异的原因之一。然而，PETHEMA模型和Mayo 2008模型之间存在较大的异质性，在独立的数据集验证中，这两个模型的一致率仅28.6％[9]。

2014年，IMWG更新了SMM的定义，梅奥诊所调整了Mayo 2008模型的界值，将同时存在sFLC比值>20％，血清M蛋白>20 g/L，骨髓克隆浆细胞比例>20％定义为高危SMM，形成了Mayo 2018模型，也就是“20-2-20”模型[10]。IMWG对Mayo 2018模型进行了验证，并在此基础上增加了FISH定义的高危细胞遗传学异常，将Mayo 2018模型的界值进行调整，形成了IMWG 2020模型，将SMM患者分为高危、中危、中低危、低危四个群体[11]。2021年一项研究在独立的数据集中同时验证了PETHEMA、Mayo 2008、Mayo 2018三个模型的一致性，结果表明，三个模型的一致性仅16.6％[12]。

2. SMM进展风险动态评估：上述模型应用的都是静态疾病负荷指标，即根据患者一次的检查结果对患者进行风险分层，然而随着时间的推移，疾病不断演进，患者肿瘤负荷可能发生变化，风险分层也可能会随之变化。研究发现，动态血清生物标志物监测有助于预测患者预后，血清M蛋白升高和血红蛋白水平下降与不良预后相关。进展型M蛋白（eMP）的定义为诊断后6个月内血清单克隆M蛋白和（或）免疫球蛋白至少增加10％（仅当M蛋白≥3 g/dl时），和（或）诊断后1年内M蛋白/免疫球蛋白至少增加25％［M蛋白至少增加0.5 g/dl和（或）免疫球蛋白增加500 mg/dl］。进展型血红蛋白的定义为诊断后1年内血红蛋白至少下降0.5 g/dl。将进展模式整合到IMWG模型或Mayo 2018模型中，能改善模型的预测价值[13]–[15]。

另一个动态预测患者预后的模型是PANGEA模型，PANGEA模型提供在线使用，不仅整合了IMWG模型中的预后因子（FLC、aBMPC、M蛋白、FISH），还加入了患者的年龄、肌酐、血红蛋白水平，更个体化地动态评估患者预后，使预后预测效能在IMWG模型的基础上增加约10％，且该模型可以不提供aBMPC结果或既往血红蛋白水平，对尚未明确诊断的SMM患者更友好，更具临床实践意义[16]。

除了构建灵敏、稳健的预后模型外，SMM预后分层另一个努力的方向是寻找更具有预后价值的分子标志物，采用创伤更小的检测方法，实现对患者的纵向评估。

3. 基因组到生物标志物预后探索的多维分析新策略：现在的模型使用的预后因子是疾病负担指标，会随着疾病的演进不断加重，只能识别有MM转化倾向和遗传学不稳定的患者[17]。越来越多的研究着眼于挖掘SMM特征性或与疾病演变相关的遗传学变化，整合二代测序（NGS）、单细胞测序和人工智能等技术，从基因组结构和序列异常到血清生物标志物，多维度识别高危SMM群体。

基因组异常的预后价值在许多研究中得以证实。研究者通过NGS识别了疾病进展时间为2.6年的高危遗传学异常[18]。此外，NGS检测患者外周血中的肿瘤细胞、监测浆细胞基因表达谱（GEP）或MYC拷贝数变异（CNA）可提示患者预后，但是该方法受限于标准化程度低和重复率低[17],[19]–[20]。单细胞RNA测序技术能够识别免疫治疗后的高危SMM患者疾病进展相关生物标志物，且能够通过外周血样本识别是否是SMM，准确率高达97％[21]。基因组不稳定性增加与MM的发病密切相关，通过骨髓中单细胞水平染色质可及性分析可以提示患者预后[22]。端粒功能障碍与基因组不稳定性相关，使用TeloView技术测量端粒的三维结构和空间组织可以量化基因组的不稳定性，从而预测患者预后，准确率高达85％[23]。

血清生物标志物也备受关注，研究者发现血清中的B细胞成熟抗原（BCMA）与疾病预后相关，通过酶联免疫吸附试验（ELISA）能够检测患者血清中的可溶性BCMA[24]。最近，高通量蛋白质组学技术被用于检测外周血中与MM及其前体疾病相关的微量蛋白质，探索其预后价值[25]。有研究致力于改善现有的评估方法，如通过人工智能评估骨髓中的肿瘤负荷，结果更可靠[26]。

尽管已经发现诸多预测预后的标志物，但这些预后标志物应用于临床尚需时间，临床应用时除了要考虑检测的灵敏度和特异度，还需考虑检测成本、通量、检测手段等问题。总之，SMM的危险度分层逐渐深入，从根据患者的疾病负担发展到根据疾病的本质特征。同时，SMM的危险度分层逐渐个体化，越来越倾向于使用动态的生物标志物和创伤更小的检测手段对疾病进展风险进行连续监测和评估。

二、高危SMM患者的治疗

1. SMM的治疗历史：上世纪90年代，美法仑和泼尼松联合方案（MP方案）治疗SMM是国际上发表的治疗SMM的初次尝试。该研究纳入50例SMM患者，研究早期治疗组（疾病诊断时治疗）和延迟治疗组（疾病进展时治疗）患者的预后。结果表明，延迟治疗组和早期治疗组的反应率和总生存期并无差异[27]。随后又有研究者尝试用MP方案治疗无症状的MM（SMM）患者，但早期应用MP方案治疗仍不能使患者获益[5],[28]。此后观察和等待策略成为SMM治疗的国际共识。2000年前后一系列治疗MM的新药上市，免疫调节剂沙利度胺广泛用于治疗MM，其衍生物来那度胺、泊马度胺在MM的治疗中表现不俗，研究者开始探索将上述新药用于治疗SMM。在比较沙利度胺和唑来膦酸联合方案（ZT方案）与唑来膦酸单药（Z）治疗高危SMM患者的研究中，双药联合治疗改善了患者的无进展生存期，但两组患者的总生存期未见差异[29]。2014年一项Meta分析比较了不同研究中SMM患者接受MP方案和ZT方案早期治疗与延迟治疗的疗效，结果表明，与延迟治疗相比，早期治疗能延缓疾病进展，但是两组的死亡率和反应率并无差异，且早期治疗带来显著的药物相关不良反应[30]。

考虑到药物治疗带来的经济负担和不良反应，推迟治疗并密切监测疾病直至患者疾病进展再开始治疗似乎对患者更有意义。然而，SMM患者的预后不一，让2年内有较高进展风险的SMM患者与低危患者一样采用观察等待方案并不合适。

SMM预后模型的发展快速推动了SMM临床研究的发展，将SMM危险度分层与新药治疗联合，针对性地治疗高危SMM患者在一定程度上改善了这部分患者的预后，但SMM患者的治疗目标尚未达成共识。如今国际上有两个主要的努力方向，一是延缓SMM进展，预防严重的终末器官损伤，二是彻底清除克隆浆细胞，在MM的前体疾病阶段治愈疾病。国际上根据延缓疾病进展或彻底治愈高危SMM两个不同的治疗目标，围绕不同抗骨髓瘤药物，开展了一系列临床试验[31]–[35]。以延缓疾病进展为目标的研究多采用较低的治疗强度，单药或双药联合治疗；而以治愈疾病为目的的研究采用与MM类似的较高治疗强度。

2. 延缓疾病进展的治疗策略：以延缓疾病进展为目标的代表性临床研究包括QuiRedex研究、E3A06研究和CENTAURUS研究。QuiRedex研究开展于2007年，首次将风险模型应用到SMM的临床研究中，开启了SMM临床研究与风险分层模型相结合的先河，此后的SMM相关的临床研究几乎都对患者进行危险度分层。QuiRedex研究纳入了西班牙PETHEMA模型和Mayo 2008模型定义的高危SMM患者，与观察组相比，来那度胺、地塞米松双药联合治疗，显著延长了高危SMM患者的疾病进展时间和总生存期[36]。该研究首次展示了早期治疗高危SMM的益处，推动了高危SMM治疗模式的转变。然而，该研究开展时间较早，患者纳入标准中未包含MRI影像学评估，可能纳入了一部分已经发生骨破坏但无明显临床症状的患者，这可能是观察组总生存期仅23个月的原因之一。另一项研究ECOG E3A06评估了来那度胺单药治疗中高危SMM的疗效，与对照组（仅观察）相比，来那度胺治疗组显著延长了患者的无进展生存期，更重要的是，该研究证实早期治疗能够显著延缓疾病进展，经过2年余，来那度胺治疗组器官损伤的发生率与观察组相比下降了72％。ECOG E3A06研究对SMM的定义更符合目前的诊断标准，然而尚未观察到总生存的获益[37]。QuiRedex研究和ECOG E3A06研究奠定了治疗高危SMM患者的基础。

CENTAURUS研究比较了不同强度达雷妥尤单抗（Dara）对患者的影响，该研究将患者分为强化治疗组、中等治疗组和短期治疗组，分别予患者4次、2次、1次Dara治疗，总缓解率（ORR）与治疗强度呈正相关，但是疗效最佳的强化治疗组ORR仅56.1％，完全缓解率不足15％，说明Dara单药可能不足以清除克隆浆细胞。在Ⅲ期AQUILA研究中，研究者延长了Dara治疗SMM的时间[33]。其他单抗如Isatuximab、Elotuzumab治疗SMM的临床研究也已展开。一项Meta分析比较了以来那度胺和单抗为基础的药物治疗高危SMM的疗效，ORR分别为59％、65％，3级以上不良反应发生率分别为36％、44％[38]。

3. 彻底治愈疾病的治疗策略：MM目前仍是一种不可治愈的疾病，若能在疾病的早期阶段，即SMM阶段彻底清除恶性克隆性浆细胞，也许能治愈MM。延缓疾病进展治疗策略不足以彻底清除克隆浆细胞，且单药治疗存在筛选耐药克隆的风险。在此基础上，研究者探索多药联合治疗高危SMM的疗效，将与治疗MM类似的方案应用到高危SMM的治疗中，研究更加强效的治疗是否能够彻底清除高危SMM患者体内的克隆浆细胞，代表性研究包括GEM-CESAR研究和ASCENT研究。GEM-CESAR研究中，患者先进行6个周期的KRd（卡非佐米+来那度胺+地塞米松）诱导治疗，序贯自体造血干细胞移植，随后进行2个周期的巩固治疗，来那度胺单药维持治疗2年，ORR为100％，最佳微小残留病阴性率为68％，随访65.8个月后，90例患者中有6例进展为MM，7例死亡。目前GEM-CESAR研究还未发表安全性评估数据[32]。ASCENT研究中，高危SMM患者接受Dara-KRd诱导治疗6个疗程、巩固治疗6个疗程，单药来那度胺维持治疗12个疗程，ORR为97％，66％的患者达到完全缓解及以上，84％患者骨髓微小残留病阴性。ASCENT研究未报道治疗相关死亡病例，但是3级以上不良反应见于52％的患者[39]。

总之，延缓疾病进展治疗策略采用较低的治疗强度，但ORR为60％左右，3级以上不良反应发生率为40％左右，而治疗强度高的治愈疾病治疗策略提示多药联合治疗能使患者实现深层次缓解，但治疗强度能否转化为总生存获益仍然未知，且多药联合治疗使不良反应发生率明显提高。目前发表的治愈疾病策略均为单臂研究，尚未表现出总生存获益，尚无切实证据证明其疗效优于以来那度胺为基础的双药或单药治疗。由此可见，高危SMM患者的治疗仍需进一步探索。国际指南中对于来那度胺治疗高危SMM态度不一，但大多推荐患者入组临床试验，寻找更多的治疗机会。除了一线抗骨髓瘤药物，双特异性抗体、CAR-T细胞等免疫疗法在高危SMM患者中的应用也在探索中，但目前仍在临床试验入组阶段（NCT05469893、NCT06100237、NCT05767359）。

经典预后模型、新型检测手段和评估方法使高危SMM患者的风险分层更加动态化、个体化。新药时代，多种免疫疗法涌现，使高危SMM患者的治疗充满了机遇与挑战，仍需进一步探索其治疗选择。
